# Progressive and Prognosis Value of Notch Receptors and Ligands in Hepatocellular Carcinoma: A Systematic Review and Meta-analysis

**DOI:** 10.1038/s41598-017-14897-6

**Published:** 2017-11-01

**Authors:** Yingshi Zhang, Dandan Li, Fan Feng, Li An, Fuhai Hui, Dasheng Dang, Qingchun Zhao

**Affiliations:** 1 Department of Pharmacy, General Hospital of Shenyang Military Area Command, Shenyang, 110840 P.R. China; 2 Department of Clinical Pharmacy, Shenyang Pharmaceutical University, Shenyang, 110016 P.R. China; 3 Research center for clinical and transitional medicine, The 302nd Hospital of Chinese PLA, Beijing, 100039 P.R. China

## Abstract

Hepatocellular carcinoma (HCC) is not sensitive to radiotherapy and chemotherapy and experiences postoperative relapse extremely easy, which is the major cause of the high mortality rate. The Notch signaling pathway is expected to become a new target for the biological treatment of HCC. We searched databases for studies that evaluated the expression of Notch receptors and/or ligands in human HCC tissue. The search yielded 15 studies that enrolled 1643 patients. Compared with non-HCC tissues, Notch 1 was associated with a higher expression level (odds risk 1.59, 95% confidence interval 0.34 to 7.45), as well as Notch 3 (2.63, 0.69 to 10.02), Notch 4 (1.33, 0.74 to 2.38) and Jagged 1 (1.47, 0.23 to 9.53); however, Notch 2 showed the opposite result (0.60, 0.30 to 1.20). Larger tumor size (>5 cm), metastasis positive, and micro vascular invasion positive were features that were associated with over-expression in Notch 1 according to the clinicopathological features. The expression levels of Notch 1, 3, 4 and Jagged 1 were associated with higher expression in HCC tissues, while Notch 2 had the opposite result. This study is registered with PROSPERO (CRD42017055782).

## Introduction

The incidence of liver cancer has been increasing in recent years. Over the past 30 years, liver cancer (mostly hepatocellular carcinoma, HCC) has mainly been prevalent in Africa and Asia. HCC is now a common disease globally and is responsible for 54% of the total number of cancer patients worldwide, with more than 600,000 related deaths estimated each year^1,2^. HCC is the fifth most common malignancy and second leading cause of cancer-related death worldwide; its 5-year survival rate is 15–17%^3^. In developing countries, HCC is the second leading cause of male cancer death, second only to lung cancer, while it ranks sixth in more developed countries^4^. In Asian and African countries, the incidence of liver cancer is associated with hepatitis B viral infection, while in Western developed countries, heavy drinking of alcohol is the main cause of liver cancer. Different inducement and influencing factors may determine the occurrence, evolution and prognosis of HCC^5,6^.

Because HCC is characterized by hidden onset, rapid development and a high degree of malignancy, early clinical diagnosis of HCC is difficult. While many patients with symptomatic treatment often have terminal cancer, most have intrahepatic metastasis and extrahepatic metastasis, liver cancer that is not sensitive radiotherapy and chemotherapy, and experience postoperative relapse extremely easy, which is the major reason for the high mortality rate^1,7^. Although the progress of various treatment modalities, including liver transplantation, in recent years has significantly improved the long-term survival of patients with HCC, the overall prognosis is still not optimistic. Therefore, early screening, early diagnosis, and initial treatment are appropriate ways to determine the prognosis of patients with liver cancer, which is important. Previous studies have shown that the occurrence, progression and pathogenesis of HCC are complex processes that involve multiple gene mutations, multiple factors and multiple pathways. Numerous related factors and molecular pathways are involved in the pathogenesis and progression of HCC. With the deepening of cell and molecular biology research of HCC, Notch signal transduction pathway disorders have been gradually recognized in the occurrence, persistence and pathological outcome of HCC.

The Notch signaling pathway is composed of Notch receptors (Notch 1–4), Notch ligands (Delta 1,3,4; Jagged 1–2) and intracellular effector molecules and is a highly conserved intercellular signal transduction pathway. The pathway affects cell growth, differentiation, proliferation and apoptosis processes, governing the development of multicellular organisms and formation of living organisms. The Notch signaling pathway is pleiotropic, and activation of this pathway not only plays an important role in the development and differentiation of normal cells but also plays an important role in the evolution of the disease, especially in corresponding tumor formation^8,9^. There is no unified understanding of the specific mechanism for the Notch signaling pathway in the formation of tumor carcinogenesis or tumor suppression. In recent years, studies on the role of the Notch signaling pathway in carcinogenesis and suppression of primary HCC have made progress. Some studies suggest that expression of Notch receptors or ligands in HCC can inhibit the proliferation of HCC cells, while the opposite view is that high expression of some Notch receptors or ligands, such as Notch 1 or Jagged1, can promote the differentiation of liver tumor and tumor vascular proliferation. The same receptor or ligand may play a specific role in promoting or inhibiting cancer in HCC according to various literature reports with different views^10,11^.

To provide new guidelines for early detection or early diagnosis, establish an individualized treatment regimen and evaluate of prognosis of HCC, this systematic review and meta-analysis observed the expression levels of Notch l–4 and Jagged l in HCC tissue, pericarcinomatous tissue and normal control tissue. We analyzed the correlation between up-regulated or down-regulated expression of the receptor and ligand in HCC tissue with respect to clinicopathological features (age, gender, tumor size, histological grade, AFP value, microvascular invasion, etc.). No previous review^12^ has provided a comprehensive overview with a meta-analysis.

## Results

### Study selection and baseline characteristics

**Figure 1:**
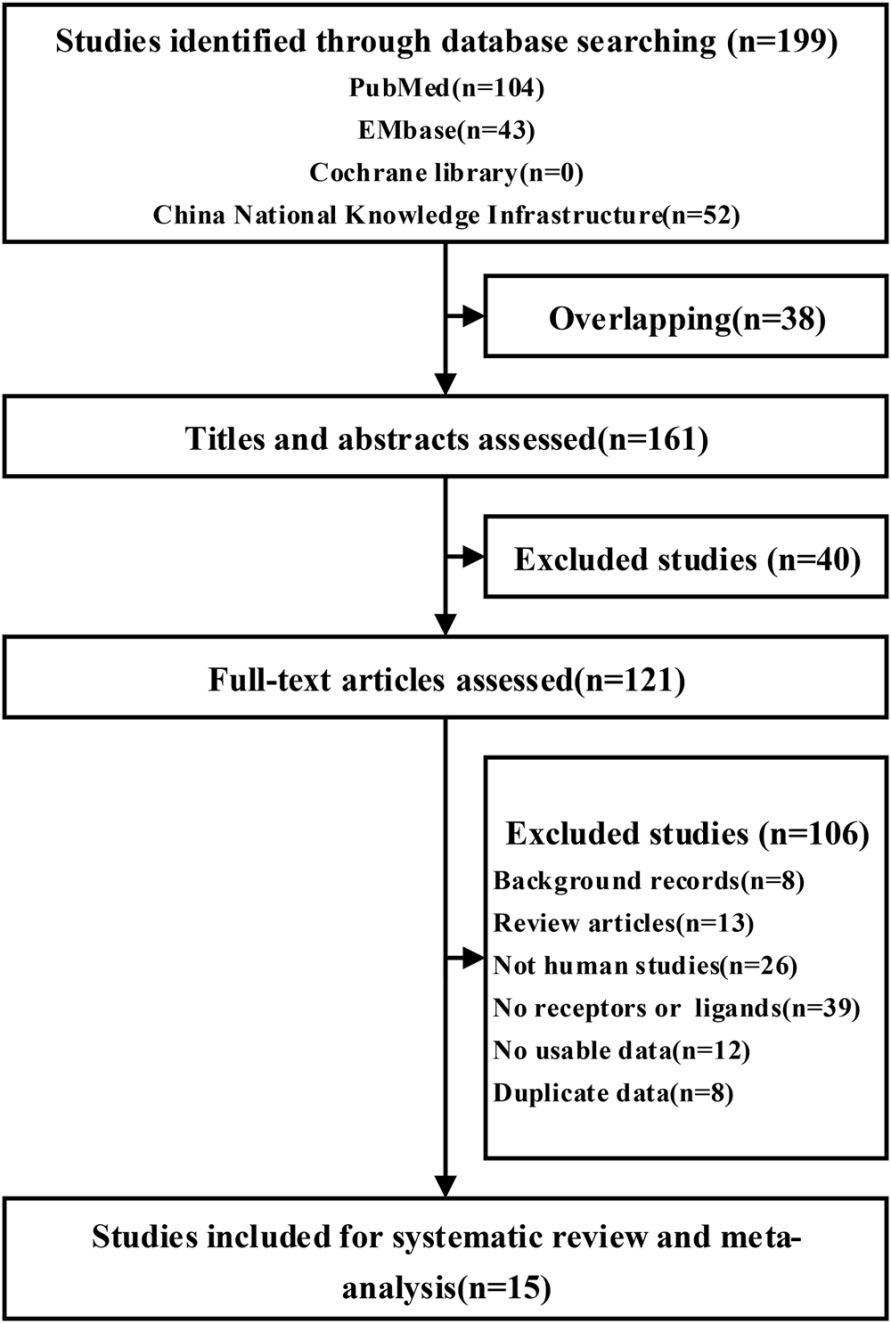
Flow of the study review process for the systematic review and meta-analysis.

Figure 1 shows the flow of studies assessed in this systematic review. Overall, fifteen studies corresponding to 1643 patients fulfilled the eligibility criteria and provided the integrated data to be contained in at least one meta-analysis^13–27^. Ten studies^14,15,17–23,25^ had Notch receptors/ligands expression data in HCC tissue with non-HCC tissue. Fourteen studies^11–18,20–25^ had additional data containing the Notch receptor and ligand expression levels with respect to molecular subtypes. Summaries of the characteristics of all of the included studies are provided in Table 1, and the differences between the mean baselines were small. The quality of the included study reports varied (range 5–7), and the required data can be assessed at an acceptable quality level. The details of the quality assessment is given in Table 1 and Table S1.

**Table 1:** Baseline Characteristics.

Reference(year)	Mean age (range)	Sample size(male/female)	Tumor stage(I-II/III-IV)	Histological grade (well/moderate/poor)	Cirrhosis(%)	HBV/HCV(%)	Metastatic(%)	Detection	Target	Follow-up(months)	Cut-off value	Quality score
Ahn S^13^	52.6(17–76)	237/51	225/63	30/195/63	50%	75.69%	6.60%	IHC	Notch 1,3,4	97.1(40–126)	Scoring of H-score 1–3 vs 4–7	5
Fang X^14^	51(11–75)	106/16	—	—	79.51%	87.70%	85.25%	RT-PCR	Jagged1	15	Scoring of H-score 0–5 vs 6–12	7
Gao J^15^	—	39/14	37/16	16/24/13	—	83.02%	—	IHC	Notch1–4, Jagged1, Delta4	—	Scoring of H-score 0–5 vs 6–12	7
Hayashi Y^16^	55.4	58/16	45/29	11/37/8	—	—	24.32%	RT-PCR	Notch1–2	—	Median expression	5
Hu L^17^	54.0 ± 9.1	78/17	73/22	12/60/21	35.79%	66.32%	14.74%	IHC	Notch 3	—	Scoring of H-score 0–5 vs 6–12	5
Liu H^18^	—	62/18	46/34	24/27/29	—	81.25%	83.75%	IHC	Notch1	—	Median H-score	7
Mi LL^19^	—	41/9	48/2	16/25/9	—	18%	22%	IHC	Notch1–4, Jagged1	—	Scoring of H-score 1–3 vs 4–7	7
Wang M^20^	49(0.9–75)	297/31	164/156	237/73/-	—	91.84%	—	IHC	Notch1, Jagged1	—	Scoring of H-score 0–5 vs 6–12	7
Wang X^21^	52(34–80)	26/9	0/35	—	—	85.71%	—	IHC	Notch 1,2,4	—	Scoring of H-score 1–3 vs 4–7	7
Yu Y^22^	50.3(41–83)	70/62	49/83	103/29	56.82%	70/62	36.36%	RT-PCR	Notch1	17(1–36)	Scoring of 0–4 vs 5–12	7
Yang Y^23^	53.8(33–72)	26/4	—	6/16/	8	—	—	46.67%	IHC	Notch1–2, Jagged1, Delta1	—	>10%
Zhang C^24^	Median 50	33/7	31/9	1/31/8	—	82.5%	35%	RT-PCR	Notch1–4, Jagged1	2–31	Median expression	5
Zhang Y^25^	66.5(48–78)	74/36	60/50	86/24	—	—	48.18%	IHC	Notch1	—	Scoring of 0–4 vs 5–12	7
Zhou L-1^26^	48.5(29–80)	74/46	32/88	41/79	—	—	31.67%	RT-PCR	Notch1	—	Scoring of 0–4 vs 5–12	5
Zhou L-2^27^	45.3(30–80)	54/32	24/62	29/57	—	—	27.91%	RT-PCR	Notch1,3	5 years	Scoring of 0–4 vs 5–12	5

HBV: hepatitis B virus; HCV: hepatitis C virus; HCC, hepatocellular carcinoma; IHC, immunohistochemistry; RT-PCR, real-time polymerase chain reaction.

### Quantitative analyses of primary outcomes

#### Comparison of Notch 1 expression in HCC and non-HCC tissues

**Figure 2:**
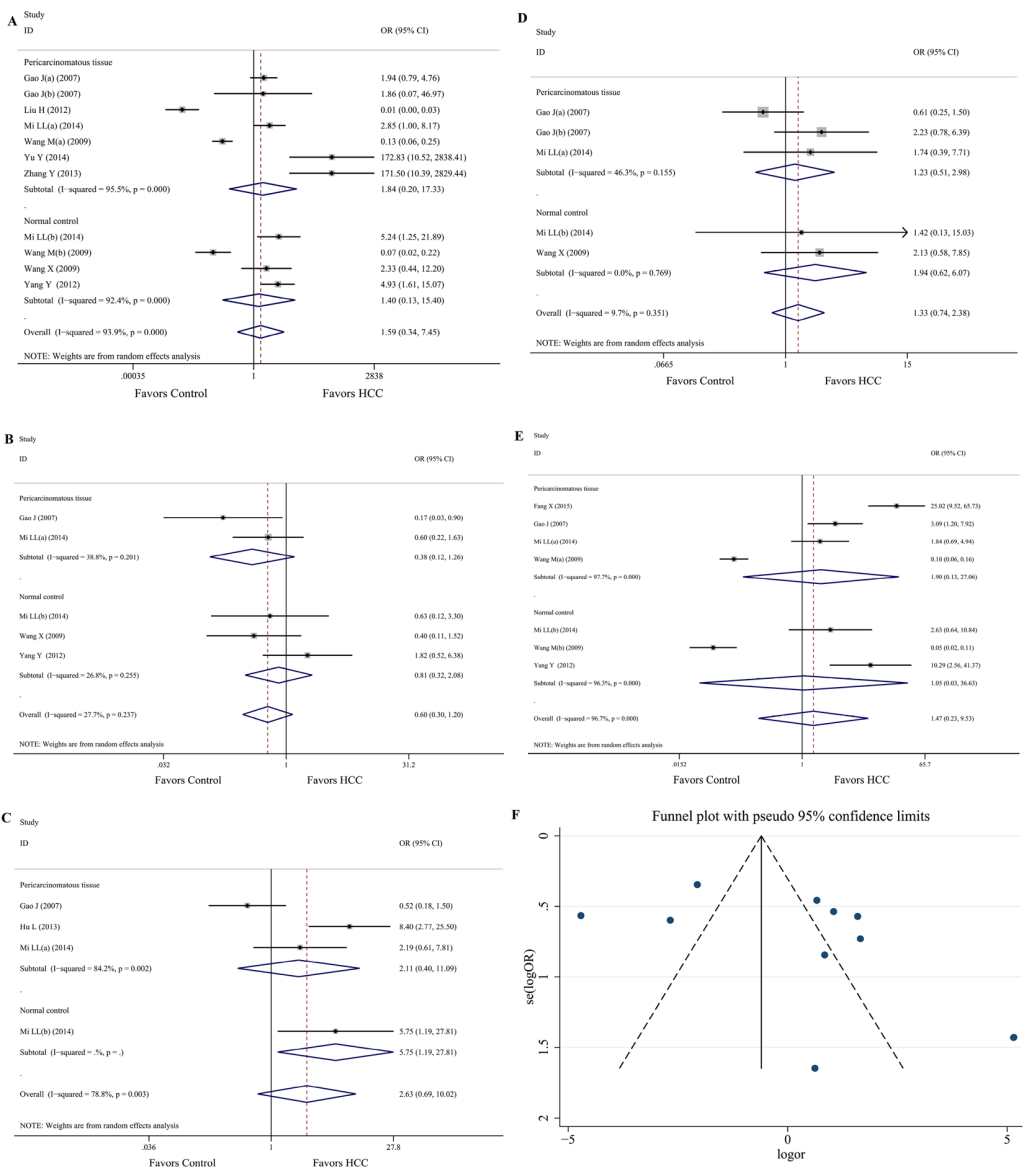
The association between the Notch receptor/ligand in HCC and non-HCC tissues. (**A**) Comparison of Notch 1 expression in HCC and non-HCC tissues. (**B**) Comparison of Notch 2 expression in HCC and non-HCC tissues. (**C**) Comparison of Notch 3 expression in HCC and non-HCC tissues. (**D**) Comparison of Notch 4 expression in HCC and non-HCC tissues. (**F**) Funnel plot of Notch 1 expression in HCC and non-HCC tissues.

Data on the differences between the two types of tissues were available from 8 studies^15,18–23,25^. Compared with pericarcinomatous tissue, Notch 1 was associated with over expression in HCC tissues (OR = 1.84, 95%CI: 0.20 to 17.33; *P* = 0.000, *I* ^2^ = 95.5%), while similar trends were found in comparison with the normal control (OR = 1.40, 95%CI: 0.13 to 15.40; *P* = 0.000, *I* ^2^ = 92.4%). Overall, 735 cases of high expression occurred in the HCC tissue group compared with 399 in the non-HCC group (OR = 1.59, 95%CI: 0.34 to 7.45; *P* = 0.000, *I* ^2^ = 93.9%; Fig. 2A), with low quality evidence according to the GRADE assessment (Table 2). Heterogeneity was moderate to high. The funnel plot showed asymmetry among our included studies, which may prove the existence of a publication bias (Fig. 2F). Moreover, no evidence of bias was identified with Begg’s test (*P* = 0.436) and Egger’s test (*P* = 0.124).

**Table 2:** Assessment of the quality of evidence using the Grading of Recommendations Assessment.

Number of analyses, by outcome	Quality assessment					Overall quality of evidence
	Risk of bias	Inconsistency	Indirectness	Imprecision	Other consideration	
**Notch 1(11)**	Very serious*	Serious^†^	Not serious	Not serious	None	++−Low* ^**†**^
**Notch 2(5)**	Very serious*	Not serious	Not serious	Not serious	None	+++−Moderate*
**Notch 3(4)**	Very serious*	Serious^†^	Not serious	Serious^‡^	None	+−Very Low* ^**†‡**^
**Notch 4(5)**	Very serious*	Not serious	Not serious	Not serious	None	+++− Moderate*
**Jagged1(7)**	Very serious*	Serious^†^	Not serious	Not serious	None	++−Low* ^**†**^

^*^All studies are case-controlled study. ^†^Substantial heterogeneity. ^‡^Small number of including.

#### Comparison of Notch 2 expression in HCC and non-HCC tissues

Compared with non-HCC tissues in four studies^15,19,21,23^, Notch 2 was associated with a decreasing tendency in HCC tissue (OR = 0.60, 95%CI: 0.30 to 1.20; *P* = 0.237, *I* ^2^ = 27.7%; Fig. 2B) with moderate quality evidence according to the GRADE assessment (Table 2). In addition, there was a similar tendency in comparison with pericarcinomatous tissue (OR = 0.38, 95%CI: 0.12 to 1.26; *P* = 0.201, *I* ^2^ = 38.8%) and the normal control (OR = 0.81, 95%CI: 0.32 to 2.08; *P* = 0.255, *I* ^2^ = 26.8%). Heterogeneity was low to moderate, and moderate evidence of bias was found with Begg’s test (*P* = 0.462) and Egger’s test (*P* = 0.527).

#### Comparison of Notch 3 expression in HCC and non-HCC tissues

Only three studies^15,17,19^ compared Notch 3 expression in HCC with non-HCC tissues. Notch 3 was associated with an increasing tendency in HCC tissues (OR = 2.63, 95%CI: 0.69 to 10.02; *P* = 0.003, *I* ^2^ = 78.8%; Fig. 2C) with very low quality evidence according to the GRADE assessment (Table 2). However, a similar tendency was found in comparison with pericarcinomatous tissue (OR = 2.11, 95%CI: 0.40 to 11.09; *P* = 0.002, *I* ^2^ = 84.2%) and the normal control (OR = 5.75, 95%CI: 1.19 to 27.81). Heterogeneity was moderate to high, with moderate evidence of bias according to Begg’s test (*P* = 0.734) and Egger’s test (*P* = 0.574).

#### Comparison of Notch 4 expression in HCC and non-HCC tissues

Compared with non-HCC tissues from three studies^14,19,21^, Notch 4 was associated with an increasing tendency in HCC tissues (OR = 1.33, 95%CI: 0.74 to 2.38; *P* = 0.351, *I* ^2^ = 9.7%; Fig. 2D), with moderate quality evidence according to the GRADE assessment (Table 2). Heterogeneity was low to moderate, and moderate evidence of bias was found according to Begg’s test (*P* = 0.734) and Egger’s test (*P* = 0.574). In addition, there was the same tendency in comparison with pericarcinomatous tissue (OR = 1.23, 95%CI: 0.51 to 2.98; *P* = 0.155, *I* ^2^ = 46.3%) and the normal control (OR = 1.94, 95%CI: 0.62 to 6.07; *P* = 0.769, *I* ^2^ = 0.0%).

#### Comparison of Jagged 1 expression in HCC and non-HCC tissues

Compared with non-HCC tissues from five studies^14,15,19,20,23^, the outcome of Jagged 1 (OR = 1.47, 95%CI: 0.23 to 9.53; *P* = 0.000, *I* ^2^ = 96.7%; Fig. 2E) did not differ between the two groups, which displayed an increasing trend in HCC tissues. In addition, there was no difference in pericarcinomatous tissue (OR = 1.90, 95%CI: 0.13 to 27.06; *P* = 0.000, *I* ^2^ = 97.7%) or the normal control (OR = 1.05, 95%CI: 0.03 to 36.63; *P* = 0.000, *I* ^2^ = 96.7%). Heterogeneity was moderate to high, with no evidence of bias according to Begg’s test (*P* = 0.368) and Egger’s test (*P* = 0.054). The quality of evidence was rated “low” according the GRADE approach (Table 2).

### Quantitative analyses of secondary outcomes

#### Correlation of Notch 1 expression and clinicopathological features

Table 3 summarizes the results of the association of the clinicopathological features with Notch 1 expression of twelve studies^13,15,16,18–20,22–27^. The results showed that the different tumor size as well as metastatic and microvascular invasion might have a large influence on the final results. Moreover, the greater possibility of a larger (>5 cm) tumor size (OR = 0.59, 95%CI: 0.41 to 0.85), positive tumor metastasis (OR = 0.42, 95%CI: 0.24 to 0.73) and positive microvascular invasion (OR = 0.53, 95%CI: 0.31 to 0.88) were linked with high Notch 1 expression in HCC. When stratifying for age, gender, histological grade, tumor stage, AFP level, Albumin level, etiology, multicentric occurrence, major portal vein invasion, liver cirrhosis and capsular invasion, no significant difference was found among the subgroups in patients with HCC. However, when grouping by gender and multicentric occurrence, the heterogeneity was low, which may suggest that these two factors are sources of heterogeneity. In conclusion, expression of Notch1 may be related to tumor size, metastasis and microvascular invasion.

**Table 3:** Notch receptor and ligand expression levels with respect to clinicopathological features.

Stratification of HCC	Notch1			Notch2			Notch3			Notch4			Jagged1		
	Studies (Subjects)	OR(95%CI)	*P, I* ^2^	Studies (Subjects)	OR(95%CI)	*P, I* ^2^	Studies (Subjects)	OR(95%CI)	*P, I* ^*2*^	Studies (Subjects)	OR(95%CI)	*P, I* ^*2*^	Studies (Subjects)	OR(95%CI)	*P, I* ^*2*^
Age(year): ≤mean vs >mean	11(1305)	1.05(0.71, 1.56)	0.010, 57.1%	3(143)	1.29(0.38, 4.42)	0.214, 35.0%	5(517)	0.89(0.37, 2.09)	0.031, 62.4%	4(423)	0.91(0.36, 2.30)	0.093, 53.3%	5(294)	0.94(0.40, 2.24)	0.078, 52.4%
Gender: Male vs Female	11(1360)	1.16(0.96, 1.40)	0.986, 0.0%	4(217)	0.76(0.13, 4.44)	0.083, 55.1%	6(612)	1.10(0.68, 1.78)	0.756, 0.0%	4(423)	1.47(0.87, 2.46)	0.775, 0.0%	5(583)	0.74(0.43, 1.30)	0.806, 0.0%
Tumor size(cm): ≤ 5 vs > 5	11(1305)	0.59(0.41, 0.85)*	0.001, 65.7%	3(143)	0.47(0.15, 1.48)	0.284, 20.5%	6(612)	0.85(0.37, 1.98)	0.004, 70.6%	4(423)	0.89(0.39, 2.00)	0.138, 45.5%	6(603)	1.10(0.73, 1.65)	0.449, 0.0%
Histological grade: Well-moderate vs Poor	12(1353)	1.65(0.84, 3.23)	0.000, 76.3%	4(199)	0.68(0.24, 1.89)	0.642, 0.0%	6(610)	0.77(0.50, 1.18)	0.510, 0.0%	4(423)	0.71(0.43, 1.17)	0.835, 0.0%	6(604)	1.51(0.73, 3.12)	0.077, 49.7%
Tumor stage: I-II vs III-IV	11(1351)	0.63(0.36, 1.11)	0.000, 83.9%	4(217)	0.39(0.16, 0.96)	0.473, 0.0%	6(612)	0.46(0.20, 1.06)	0.029, 59.8%	4(423)	1.17(0.48, 2.88)	0.157, 42.4%	5(452)	2.24(1.57, 3.20)	0.365, 7.3%
AFP level(ng/mL): ≤mean vs> mean	9(1165)	0.95(0.57, 1.58)	0.001, 68.7%	2(90)	1.13(0.45, 2.82)	0.652, 0.0%	4(453)	1.15(0.60, 2.21)	0.225, 31.1%	3(359)	3.24(2.01, 5.21)	0.367, 0.2%	5(552)	1.29(0.53, 3.14)	0.011, 69.5%
Albumin level (g/dL): ≤mean vs > mean	1(288)	0.85(0.40, 1.81)	—	—			1(288)	1.74(0.75, 4.03)	—	1(286)	1.16(0.53,2.55)	—	1(288)	0.85(0.40, 1.81)	—
Etiology: Non-viral vs HBV/HCV	7(920)	1.33(0.57, 3.09)	0.006, 69.2%	2(98)	0.69(0.18, 2.63)	0.194, 40.6%	4(473)	1.32(0.76, 2.28)	0.804, 0.0%	3(370)	0.75(0.41, 1.40)	0.895, 0.0%	4(533)	1.34(0.69, 2.61)	0.841, 0.0%
Metastatic: Negative vs Positive	7(838)	0.42(0.24, 0.73)*	0.032, 56.6%	2(132)	0.35(0.06, 2.08)	0.042, 75.7%	4(414)	0.58(0.21, 1.64)	0.042, 68.2%	2(320)	2.13(1.27, 3.56)	0.986, 0.0%	2(162)	0.58(0.08, 4.04)	0.116, 59.5%
Multicentric occurrence: Negative vs Positive	6(786)	0.80(0.55, 1.18)	0.382, 5.4%	1(50)	1.25(0.27, 5.73)	—	4(519)	0.64(0.19, 2.19)	0.094, 53.1%	1(288)	0.37(0.12, 1.16)	—	2(172)	1.07(0.40, 2.86)	0.400, 0.0%
Major portal vein invasion: Negative vs Positive	6(985)	0.59(0.24, 1.43)	0.000, 77.4%	1(50)	1.32(0.22, 7.87)	—	4(519)	0.52(0.20, 1.36)	0.123, 48.0%	2(338)	0.90(0.31, 2.64)	0.914, 0.0%	1(50)	2.47(0.39, 15.73)	—
Microvascular invasion: Negative vs Positive	2(420)	0.53(0.31, 0.88)*	0.223, 32.6%	—			1(288)	0.66(0.36, 1.18)	—	1(298)	1.83(1.14, 2.92)	—	—		
Liver cirrhosis: Negative vs Positive	2(422)	1.03(0.45, 2.38)	0.044, 75.3%	—			2(383)	0.68(0.41, 1.11)	0.839, 0.0%	1(288)	1.00(0.63, 1.60)	—	1(122)	0.74(0.31, 1.77)	—
Capsular invasion: Negative vs Positive	1(50)	0.91(0.17, 5.03)	—	1(50)	1.40(0.38, 5.20)	—	2(146)	1.39(0.58, 3.34)	0.657, 0.0%	1(50)	1.92(0.16,22.61)	—	2(171)	0.81(0.40, 1.65)	0.867, 0.0%

AFP, α-fetoprotein; HCC, hepatocellular carcinoma; *Result with significant differences.

#### Correlation of other components of Notch signaling pathway expression and clinicopathological features

When stratifying for tumor size, metastasis, microvascular invasion, among other factors, no significant differences were found among the subgroups in Notch 2 expression in patients with HCC^15,16,19,24^. No difference in Notch 3 expression^13,15,17,19,24,26^ was found between clinicopathological features, whereas no difference when stratifying for tumor size, metastasis, microvascular invasion, among others factors was observed for Notch 4 expression^11,13,17,22^. Jagged 1 expression^14,15,19,20,23,24^ also had no significant difference among the clinicopathological features (Table 3).

## Discussion

We identified data from 15 studies that enrolled 1643 patients with HCC. We found that Notch 1, Notch 3, Notch 4 and Jagged 1 were associated with higher expression levels in HCC tissues compared with non-HCC tissues (both pericarcinomatous tissues and normal control; Fig. 2A,C–E), while Notch 2 was associated with lower expression levels (Fig. 2B). With moderate to high heterogeneity between the studies, low to moderate quality evidence was assessed by GRADE. According to the results of the Notch receptor and ligand expression levels with respect to clinicopathological features, larger tumor size (>5 cm), metastasis positive, micro vascular invasion positive were associated with overexpression of Notch 1(Table 3).

HCC is a joint malignant tumor. The molecular mechanisms and pathways of HCC have been shown to be involved in the progression of HCC in a wide range of studies. Numerous studies have shown that Notch signaling plays a very important role in carcinogenesis, progression, invasion and neovascularization in many types of human tumors^28,29^. However, until now, the role of Notch receptors in hepatocellular carcinoma has been less studied and the function and expression of Notch in the development of normal liver and liver diseases have been extensively studied^30^. Some studies have shown that the Notch signaling pathways have a negative impact on liver cell growth and proliferation, while there are also reports that show that the Notch signaling pathways promote liver cell growth and proliferation^31^. For example, in a rat model of liver damage or liver regeneration, expression of Notch l, Notch 2, Notch 3, Delta l and Jagged l in hepatocytes is increased^32^. After partial hepatectomy in animal model rats, the Notch l and Jagged l proteins were regulated and Notch l ICN was increased. The Notch l/Jagged l signaling pathway is activated during liver regeneration^33^. This study then was important to research on HCC.

The results of our meta-analysis are that Notch 1, 3, 4 and Jagged 1 are overexpressed in HCC tissue, while Notch 2 expression is reduced, and some clinical studies have also reached similar results^34,35^. However, there was no significant difference among all of the primary outcomes. The authors speculated that this may be due to the similarities between liver cancer and cervical cancer; Notch l in the early stages of cervical cancer promotes cancer, but showed anti-cancer effects in the late stages^36^. This may also lead to heterogeneity between studies. Moreover, Weijzent’s experiments using human embryonic kidney epithelial cells found that Notch l can induce Notch 4, proving that Notch l has synergy with Notch 4^37^; Shimizut, in rat experiments, demonstrated that Notch 2 inhibits Notch l and Notch 3, suggesting that Notch 2 has a negative correlation with Notch l and Notch 3^38^. These above studies also support our results.

This review has some limitations that are typical of meta-analyses without access to individual patient’s data, including not being able to control for differences between the included studies. Moreover, the HCC clinical stage was variable. Moreover, our analyses are stratified by pericarcinomatous tissue and normal control tissue, which is an aggregated variable and opens our results to potential bias. Moreover, the funnel plot (Fig. 2F) may indicate the existence of a publication bias, while both Begg’s test and Egger’s test did not reach similar. This may reveal that there is a moderate publication bias in our meta-analysis. In addition, the majority of our included studies were conducted in Asia, probably because Asia and Africa have a greater incidence of liver cancer, which led to certain geographical limitations. Additionally, different receptor/ligand detection methods (RT-PCR and IHC) could also lead to differences. One way to reduce this risk of bias would be to define the grouping of the original studies, provide patient data for each, and expand the scope of the studies to the global scale. In our included studies, no randomization or blinding was performed, the quality was not high, and the study level was only sufficient for meta-analysis.

This is the first systematic review to specifically address the Notch receptor and ligand for patients with HCC. Compared with the most recent meta-analysis^12^ on this topic, which had similar inclusion criteria, we increased the number of receptors and ligands of the quantitative analyses and analyzed the Notch receptor and ligand expression levels with respect to clinicopathological features.

Early diagnosis of AFP, TPA, AFU and so on in a series of tumor markers (in fact, the majority of patients in the middle or late stage of cancer) cannot be timely and accurate. There is a large limitation on the accuracy of clinical prognosis. Thus, it is regrettable that the world has still not yet found a way to truly respond to the needs of clinical or basic research for the sensitive, specific, simple and easy detection of HCC markers. Therefore, we continue to seek specific markers of HCC at the molecular level to serve as prognostic indicators for patients and indicators to guide clinical treatment, which has become the focus of attention of current basic medical and clinical research. Some studies have studied Notch receptor ligand binding interference, receptor activation and inhibition of NICD nuclear complex formation as well as the inhibition of $\gamma$-secretase as ways of treating tumors^10^. It was concluded that the Notch signaling pathway may serve as a new target for the biological treatment of HCC.

This systematic review and meta-analysis of available evidence suggests that for patients with HCC, the expression levels of Notch 1, 3, 4 and Jagged 1 are associated with higher expression in HCC tissues, while Notch 2 has the opposite result. Larger tumor size (>5 cm), metastasis positive, micro vascular invasion positive features were associated with an increase in Notch 1 expression according to clinicopathological features. In addition, because only a small proportion of studies was analyzed, high quality studies must be undertaken in patients at the global scale to validate our results.

## Methods

This review was performed using a prospective protocol. It was performed according to the Preferred Reporting Items for Systematic Reviews and Meta-analyses (PRISMA) statement^39^. The project was prospectively registered with the PROSPERO database of systematic reviews, number CRD 42017055782^40^.

### Eligibility Criteria

We evaluated all studies that compared the expression level of HCC tissue with non-HCC tissue and excluded studies that did not provide comparative or missing outcomes. Studies of the differences between the clinicopathological features of HCC tissue in each individual study were accepted. We did not restrict our search by language, date, or publication status. During February 2017, we searched PubMed, Embase, the Cochrane library and China National Knowledge Infrastructure of case-controlled studies using the MeSH terms “hepatocellular carcinoma” and “notch receptor/ligand”. These studies were explored and combined (see details in Supplementary Table 2). We also browsed reference lists. Studies were eligible for inclusion if they evaluated the expressions of Notch receptors and/or ligands in human HCC tissue. We considered studies including Notch receptors and/or ligands expression evaluated in HCC tissues. Notch signaling expressions were considered by immunohistochemistry (IHC) or real-time reverse transcription polymerase chain reaction (RT-PCR). If multiple eligible studies contained significant overlap in patients, the largest one of them were employed in our meta-analysis.

### Selection of Studies and Data Extraction

Two of the authors (ZYS and LDD) assessed the eligibility of the articles found in the literature search. Both independently checked the abstracts and full-texts for eligibility and resolved any disagreements by discussion with the third reviewer (ZQC). Data were extracted into specially designed tables. Study characteristics, such as the age, sample size, tumor stage, histological grade, cirrhosis (%), HBV/HCV (%), metastatic (%), detection, target, follow-up, and cut-off value, were recorded. For studies that included Notch signaling expressions both with and without HCC tissue, we ascertained the difference between two types of tissue as the primary outcome. Whether expression of non-HCC tissues was reported, we also explored the association between the reported difference and age, gender, tumor size (cm), histological grade, tumor stage, AFP level (ng/mL), Albumin level (g/dL), etiology, metastasis, multicentric occurrence, major portal vein invasion, microvascular invasion, liver cirrhosis, and capsular invasion in HCC tissues as the secondary outcome.

### Risk of a Bias Assessment and the Grading Quality of Evidence

Two of the authors evaluated the risk of bias in each study included in this meta-analysis using the Newcastle-Ottawa Scale (NOS) for assessing the risk of bias. NOS^41^ was used as suggested by the Cochrane Non-Randomized Studies Methods Working Group. NOS utilized the following criteria labels, “yes” or “no”, for the following questions: Is the case definition adequate? Representativeness of the cases? Selection of Controls? Definition of Controls? Comparability of cases and controls? (0–2) Ascertainment of exposure? (0–2) Same method of ascertainment for cases and controls? and nonresponse rate? We excluded some studies which scores less than 5.

We assessed the quality of the evidence for our primary outcomes according to the Grading of the Recommendations Assessment, Development and Evaluation (GRADE) system using GRADEpro GDT^42,43^. The GRADE system assesses the risk of bias (study limitations), imprecision, inconsistency, indirectness of the study results, and publication bias (classifying each as high, moderate, low, or very low) across the body of evidence to derive an overall summary of the quality of the evidence.

### Data synthesis and analysis

All statistical analyses were performed using StataMP statistical software (version 14, Stata Co. College Station, TX, United States). Meta-analysis was undertaken where data were abundant. Where data were available, we calculated the odds ratio (OR) with 95% confidence intervals (CI) for each outcome in each study, and synthesized the outcomes using a random model. We chose the random effects model over the fixed effects model because we anticipated that there would be substantial clinical and methodological heterogeneity. We evaluated the statistical heterogeneity of the expression values between the studies using the χ^2^ test. Statistical heterogeneity was also tested by *I* ^2^, with an *I* ^2^ < 25% indicating low heterogeneity^44^, and we also defined the heterogeneity of *I* ^2^ > 50% as moderate and >75% as substantial. We defined studies reporting multiple comparators as sub-studies (mark as a/b) to avoid double counting and mistreating the data. Publication bias was measured with funnel plots, Begg’s test and Egger’s test, and a two-tailed value of *P* = 0.05 was considered significant for the latter two tests.

### Patient involvement

No patients were involved in setting the research question or the outcome measures, nor were they involved in developing plans for the design or implementation of the study. No patients were asked to advise on interpretation or writing up of results. There are no plans to disseminate the results of the research to study participants or the relevant patient community.

## Electronic supplementary material

Supplementary Information
