# Supplementary material for: Progressive and Prognosis Value of Notch Receptors and Ligands in Hepatocellular Carcinoma: A Systematic Review and Meta-analysis
Source: Sci Rep. 2017 Nov 1;7:14809. doi: 10.1038/s41598-017-14897-6 (PMC5665870; doi:10.1038/s41598-017-14897-6)
Supplement: Supplementary file 1 — Supplementary Information [file 41598_2017_14897_MOESM1_ESM.doc]

# Progressive and Prognosis Value of Notch Receptors and Ligands in Hepatocellular Carcinoma: A Systematic Review and Meta-analysis

Yingshi Zhang, PhD^1^; Dandan Li, MD^1^; Fan Feng, PhD^3^; Li An, MD^1^; Fuhai Hui, PhD^1^; Dasheng Dang, PhD^1,2*^; Qingchun Zhao, PhD^1,2,*^

^1^Department of Clinical Pharmacy, Shenyang Pharmaceutical University, Shenyang, 110016, P.R. China

^2^Department of Pharmacy, General Hospital of Shenyang Military Area Command, Shenyang, 110840, P.R. China

^3^Research center for clinical and transitional medicine, The 302nd Hospital of Chinese PLA, Beijing 100039, P.R. China

***The First Corresponding Author**

**Qingchun Zhao**

Department of Clinical Pharmacy, Shenyang Pharmaceutical University

No. 103 Wenhua Road, Shenyang, 110840, China.

E-mail: [z](mailto:lqyxm@hotmail.com)haoqingchun1967@163.com (Q. Zhao).

***The Second Corresponding Author**

**Dasheng Dang**

Department of Clinical Pharmacy, Shenyang Pharmaceutical University

No. 103 Wenhua Road, Shenyang, 110840, China.

E-mail: dds-sy@163.com (D. Dang).

**Supplementary Table 1. Risk of Bias Assessments**

Risk of Bias Assessment using the Newcastle-Ottawa Scale for Case-control Studies


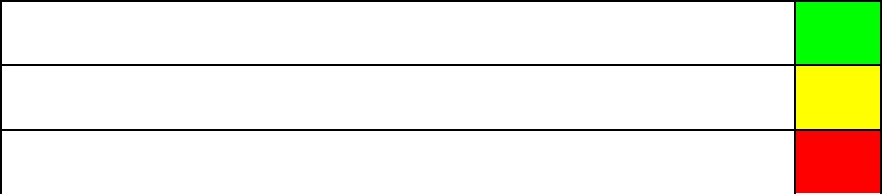


Low Risk of Bias

Intermediate or Unknown Risk of Bias

High Risk of Bias

| **Study** | **Is the case definition adequate** | **Representativeness of the cases** | **Selection of Controls** | **Definition of Controls** | **Comparability of cases and controls (/2)** | **Ascertainment of exposure(/2)** | **Same method of ascertainment for cases and controls** | **Non-Response rate** | **Overall rating and TOTAL SCORE / 10** |
| --- | --- | --- | --- | --- | --- | --- | --- | --- | --- |
|  |  |  |  |  |  |  |  |  |  |
| Ahn S (2013)1 | 1 | 1 | 0 | 0 | 1 | 0 | 1 | 1 | | | 5 | | --- | | | --- | --- | |
| Fang X (2015)2 | 1 | 1 | 0 | 1 | 2 | 0 | 1 | 1 | 7 |
| Gao J (2007)3 | 1 | 1 | 0 | 1 | 2 | 0 | 1 | 1 | 7 |
| Hayashi Y (2015)4 | 1 | 1 | 0 | 0 | 1 | 0 | 1 | 1 | | 5 | | --- | |
| Hu L (2013)5 | 1 | 1 | 0 | 0 | 1 | 0 | 1 | 1 | | 5 | | --- | |
| Liu H (2012)6 | 1 | 1 | 0 | 1 | 2 | 0 | 1 | 1 | 7 |
| Mi LL (2014)7 | 1 | 1 | 0 | 1 | 2 | 0 | 1 | 1 | 7 |
| Wang M (2009)8 | 1 | 1 | 0 | 1 | 2 | 0 | 1 | 1 | 7 |
| Wang X (2009)9 | 1 | 1 | 0 | 1 | 2 | 0 | 1 | 1 | 7 |
| Yu Y (2014)10 | 1 | 1 | 0 | 1 | 2 | 0 | 1 | 1 | 7 |
| Yang Y (2012)11 | 1 | 1 | 0 | 1 | 2 | 0 | 1 | 1 | 7 |
| Zhang C (2011)12 | 1 | 1 | 0 | 0 | 1 | 0 | 1 | 1 | | 5 | | --- | |
| Zhang Y (2013)13 | 1 | 1 | 0 | 1 | 2 | 0 | 1 | 1 | 7 |
| Zhou L-1 (2013)14 | 1 | 1 | 0 | 0 | 1 | 0 | 1 | 1 | | 5 | | --- | |
| Zhou L-2 (2013)15 | 1 | 1 | 0 | 0 | 1 | 0 | 1 | 1 | | 5 | | --- | |

**Supplementary Table 2 Search strategies for Pubmed, EMBASE and the Cochrane Library database.**

**Search strategies for PubMed**

#1. ("carcinoma, hepatocellular"[MeSH Terms] OR ("carcinoma"[All Fields] AND "hepatocellular"[All Fields]) OR "hepatocellular carcinoma"[All Fields] OR ("hepatocellular"[All Fields] AND "carcinoma"[All Fields])) AND notch[All Fields] AND ("Receptor"[Journal] OR "receptor"[All Fields])

#2. HCC[All Fields] AND notch[All Fields] AND ("Receptor"[Journal] OR "receptor"[All Fields])

#3. 1 and 2

#4. ("carcinoma, hepatocellular"[MeSH Terms] OR ("carcinoma"[All Fields] AND "hepatocellular"[All Fields]) OR "hepatocellular carcinoma"[All Fields] OR ("hepatocellular"[All Fields] AND "carcinoma"[All Fields])) AND notch[All Fields] AND ("ligands"[MeSH Terms] OR "ligands"[All Fields] OR "ligand"[All Fields])

#5. HCC[All Fields] AND notch[All Fields] AND ("ligands"[MeSH Terms] OR "ligands"[All Fields] OR "ligand"[All Fields])

#6.4 and 5

#7.("carcinoma, hepatocellular"[MeSH Terms] OR ("carcinoma"[All Fields] AND "hepatocellular"[All Fields]) OR "hepatocellular carcinoma"[All Fields] OR ("hepatocellular"[All Fields] AND "carcinoma"[All Fields])) AND notch1[All Fields]

#8. HCC[All Fields] AND notch1[All Fields]

#9.7 and 8

#10.("carcinoma, hepatocellular"[MeSH Terms] OR ("carcinoma"[All Fields] AND "hepatocellular"[All Fields]) OR "hepatocellular carcinoma"[All Fields] OR ("hepatocellular"[All Fields] AND "carcinoma"[All Fields])) AND notch2[All Fields]

#11. HCC[All Fields] AND notch1[All Fields]

#12. 11and 12

#13.("carcinoma, hepatocellular"[MeSH Terms] OR ("carcinoma"[All Fields] AND "hepatocellular"[All Fields]) OR "hepatocellular carcinoma"[All Fields] OR ("hepatocellular"[All Fields] AND "carcinoma"[All Fields])) AND notch3[All Fields]

#14.HCC[All Fields] AND notch3[All Fields]

#15. 13 and 14

#16. ("carcinoma, hepatocellular"[MeSH Terms] OR ("carcinoma"[All Fields] AND "hepatocellular"[All Fields]) OR "hepatocellular carcinoma"[All Fields] OR ("hepatocellular"[All Fields] AND "carcinoma"[All Fields])) AND notch4[All Fields]

#17. HCC[All Fields] AND notch4[All Fields]

#18. 16 and 17

#19. ("carcinoma, hepatocellular"[MeSH Terms] OR ("carcinoma"[All Fields] AND "hepatocellular"[All Fields]) OR "hepatocellular carcinoma"[All Fields] OR ("hepatocellular"[All Fields] AND "carcinoma"[All Fields])) AND jadded1[All Fields]

#20.HCC[All Fields] AND jadded1[All Fields]

#21. 19 and 20

#22. 6 OR 9 OR 12 OR 15 OR 18 OR 21

**Search strategies for EMbase**

#1 ‘hepatocellular carcinoma$’:ab,ti

#2 ‘HCC$’:ab,ti

#3 ‘notch receptor$’:ab,ti

#4 ‘notch ligand$’/exp

#5 ‘notch 1$’:ab,ti

#6 ‘notch 2’:ab,ti

#7 ‘notch 3’:ab,ti

#8 ‘notch 4’:ab,ti

#9 ‘jadded 1’:ab,ti

#10 #1 OR #2 OR #3 OR #4 OR #5 OR #6 OR #7 OR #8 OR #9

**Search strategies for Cochrane library**

#1.hepatocellular carcinoma and HCC, notch receptor and notch ligand, notch 1 or notch 2 or notch 3 or notch 4 OR jadded 1.

#2. MeSH descriptor
